# MicroRNA‐29c‐tetrahedral framework nucleic acids: Towards osteogenic differentiation of mesenchymal stem cells and bone regeneration in critical‐sized calvarial defects

**DOI:** 10.1111/cpr.13624

**Published:** 2024-02-27

**Authors:** Jiafei Sun, Xingyu Chen, Yunfeng Lin, Xiaoxiao Cai

**Affiliations:** ^1^ State Key Laboratory of Oral Diseases, National Center for Stomatology, National Clinical Research Center for Oral Diseases, West China Hospital of Stomatology Sichuan University Chengdu China; ^2^ Sichuan Provincial Engineering Research Center of Oral Biomaterials Chengdu Sichuan China

## Abstract

Certain miRNAs, notably miR29c, demonstrate a remarkable capacity to regulate cellular osteogenic differentiation. However, their application in tissue regeneration is hampered by their inherent instability and susceptibility to degradation. In this study, we developed a novel miR29c delivery system utilising tetrahedral framework nucleic acids (tFNAs), aiming to enhance its stability and endocytosis capability, augment the efficacy of miR29c, foster osteogenesis in bone marrow mesenchymal stem cells (BMSCs), and significantly improve the repair of critical‐sized bone defects (CSBDs). We confirmed the successful synthesis and biocompatibility of sticky ends‐modified tFNAs (stFNAs) and miR29c‐modified stFNAs (stFNAs‐miR29c) through polyacrylamide gel electrophoresis, microscopy scanning, a cell counting kit‐8 assay and so on. The mechanism and osteogenesis effects of stFNAs‐miR29c were explored using immunofluorescence staining, western blotting, and reserve transcription quantitative real‐time polymerase chain reaction. Additionally, the impact of stFNAs‐miR29c on CSBD repair was assessed via micro‐CT and histological staining. The nano‐carrier, stFNAs‐miR29c was successfully synthesised and exhibited exemplary biocompatibility. This nano‐nucleic acid material significantly upregulated osteogenic differentiation‐related markers in BMSCs. After 2 months, stFNAs‐miR29c demonstrated significant bone regeneration and reconstruction in CSBDs. Mechanistically, stFNAs‐miR29c enhanced osteogenesis of BMSCs by upregulating the Wnt signalling pathway, contributing to improved bone tissue regeneration. The development of this novel nucleic acid nano‐carrier, stFNAs‐miR29c, presents a potential new avenue for guided bone regeneration and bone tissue engineering research.

## Graphical abstract

In this research, we employed sticky ends modified‐tetrahedral framework nucleic acids (stFNAs) as a delivery system for miR29c, referred to as stFNAs‐miR29c. In previous studies, stFNAs have exhibited remarkable capabilities in terms of drug‐loading, endocytosis capacity and stability. Through the use of stFNAs‐miR29c, we effectively addressed the challenges associated with the instability of miR29c and its limited endocytosis ability. Thus, this delivery system improved the efficiency of miR29c transportation and enhanced its biological function. By activating the Wnt pathway, stFNAs‐miR29c successfully increased the expression of RUNX2 and other factors specific to osteogenesis. This process benefited to the increased deposition of mineralised nodules and optimal bone regeneration in critical‐sized bone defects. These findings have significant implications for the broader applications of stFNAs‐miR29c in long‐term tissue regeneration and reconstruction.

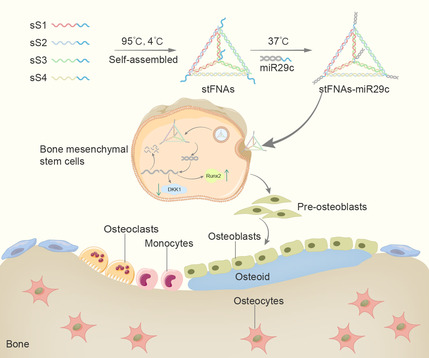

## 1 INTRODUCTION

The prevalence of fractures or bone defects caused by trauma, tumours, infections, osteomyelitis, and osteoporosis is rising annually due to the global increase in ageing populations.^1^, ^2^ While autogenous bone grafting is the clinically accepted gold standard for bone defect repair, it faces considerable challenges and limitations due to the irregular shapes and varied locations of defects, constrained donor bone volume and high technical demands.^3^ Other graft materials such as allografts and xenografts are less preferred due to ethical concerns, species limitations, potential disease transmission risks and often inadequate fit.^4^ In bone tissue engineering, the use of scaffold materials that incorporate osteogenic differentiation activators and/or seed cells is becoming more prevalent; however, issues with unstable functional groups during scaffold construction, the use of cytotoxic agents, and variable coupling efficiencies can negatively impact material biocompatibility and their subsequent applications. Thus, developing biomaterials that are simple to synthesise, biocompatible and effective in modulating the osteogenic differentiation of host cells is increasingly challenging yet promising.

Based on the Watson–Crick base complementary pairing principle, DNA is a versatile polymer. Numerous studies have demonstrated its utility in assembling and synthesising various nanomaterials with diverse morphologies and structures.^5^, ^6^, ^7^, ^8^, ^9^, ^10^ Owing to its excellent biocompatibility, biodegradability, and gene editing capabilities, the DNA nano nucleic acid system has been widely explored for applications in drug tracking,^11^ cell transmission^12^ and tissue engineering.^13^ Among these, tetrahedral framework nucleic acids (tFNAs), a representative DNA nanostructure, have become significant research focal points in the nanomaterials field due to their outstanding properties.^14^, ^15^ The self‐assembly and microscopic tetrahedral configuration of tFNAs confer excellent editability and stability, beneficial for disease early diagnosis, prognosis and treatment planning.^16^, ^17^ Furthermore, numerous studies have shown that tFNAs possess high cell entry efficiency and low biotoxicity,^18^, ^19^, ^20^, ^21^ greatly expanding the potential applications of this nanomaterial. Current research has confirmed the efficacy of tFNAs in promoting tissue regeneration in skin,^22^ nerve^23^ and joint^24^ injuries.

MicroRNA, a type of endogenous non‐coding small RNA molecule, plays diverse roles in gene regulation, cell differentiation, tissue regeneration, biological development and disease pathogenesis.^25^, ^26^ Among these, miR29c, a member of the miR29 family, activates the Wnt signalling pathway and promotes osteogenesis of bone marrow mesenchymal stem cells (BMSCs). It achieves this by downregulating the expression of Dickkopf‐associated protein (DKK1) and inhibiting the effects of the LRP5/6 inhibitory receptors on the Wnt pathway.^27^, ^28^, ^29^ Current miRNA therapies involve synthesising exogenous miRNA analogues or inhibitors (miRNA‐mimics/inhibitors) to mimic or inhibit endogenous miRNA functions. In this study, miR29c‐mimics were synthesised to downregulate DKK1 expression, activate the Wnt pathway, upregulate downstream factors such as runt‐related transcription factor 2 (RUNX2), and ultimately promote local osteogenic differentiation. However, miR29c‐mimics often degrade, and existing miRNA delivery systems frequently encounter issues with biosafety and delivery efficiency.^25^, ^27^, ^29^ Addressing these challenges to develop an efficient and safe miRNA delivery system is a pressing issue. In recent years, scholars have gradually devoted themselves to exploring the role of tFNAs as a nanoparticle carrier for miRNAs and achieved good results.^30^

In this study, we synthesised tFNAs with sticky ends (stFNAs) by attaching specially designed sticky ends to the four vertices of tFNAs. Then, stFNAs were incubated with exogenous miR29c‐mimics (referred to as miR29c) and synthesised into stFNAs‐miR29c‐mimics (referred to as stFNAs‐miR29c) through sticky terminal base complementary pairing. The stability and endocytosis efficiency of stFNAs facilitated the delivery of miR29c to target cells, overcoming miR29c's instability and improving its intracellular efficiency.

## 2 MATERIALS AND METHODS

### 2.1 Design and synthesis of stFNAs and stFNAs‐miR29c

Four DNA single strands (sS1.sS2, sS3 and sS4) and miR29c with sticky ends were synthesised by Sangon (Shanghai). These ssDNAs were combined in equimolar mass ratios in TM buffer and thoroughly mixed. The mixture was heated in a thermocycler to form stFNAs nanoparticles. The TM buffer contained a 10 mM concentration of Tris and a 5 mM concentration of MgCl_2_. The miR29c was proportionally mixed with stFNAs and incubated at a constant temperature of 37°C for 30 min to ensure successful modification of stFNAs‐miR29c. Notably, miR29c was mixed with stFNAs at a 5:1 ratio, enriching the system with miRNA and ensuring the modification of all four vertices of stFNAs with miRNAs. The sequences of ssDNA and miR29c are listed in Supporting Information, Table S1.

### 2.2 Identification and characterisation of stFNAs and stFNAs‐miR29c

We confirmed the successful synthesis of stFNAs and stFNAs‐miR29c using polyacrylamide gel electrophoresis (PAGE) and capillary electrophoresis (CE, Qsep100, Bioptic). The tetrahedral structure of stFNAs and the small tail‐like structure of miR29c were verified using transmission electron microscopy (TEM, Hitachi Ltd.) and atomic force microscopy (AFM, Shimadzu). Finally, we determined the particle size and ζ potential of stFNAs and stFNAs‐miR29c using dynamic light scattering (DLS, Nano ZS).

### 2.3 Cell culture

BMSCs were isolated from the bone marrow cavities of neonatal rats. We acquired 2‐week‐old rats from Ensiweier Co., Ltd., and harvested primary BMSCs using enzymatic digestion after rinsing. The chosen culture medium concluded with basic alpha medium supplemented, bovine serum and antibiotics. Upon reaching 80%–90% confluency, BMSCs were passaged using trypsin solution. Excess cells were cryopreserved in liquid nitrogen and thawed as needed.

### 2.4 Sample grouping

In both in vivo and in vitro experiments, samples were categorised into four groups: the Ctrl, stFNAs, stFNAs‐NC and stFNAs‐miR29c groups. For the in vitro Ctrl group, an equivalent volume of TM buffer was added to the medium with 3% serum concentration to negate the impact of this salt solution on the cells. The stFNAs‐NC group was synthesised as a nonsensical control with a scrambled sequence (NC) at the four endpoints of stFNAs, designed to replicate the spatial configuration of stFNAs‐miR29c and mitigate the impact of its three‐dimensional structure on BMSCs. The stFNAs‐NC group was established to bolster the validity of the experimental group's positive outcomes and minimise extraneous influences, notably the effects of stFNAs' unique spatial arrangement when carrying the nucleic acid sequence on target cells and the local microenvironment.

### 2.5 Cellular uptake of miR29c and stFNAs‐miR29c

To assess the cell uptake of miR29c and stFNAs‐miR29c, we examined the intracellular fluorescence intensity of these nanomaterials using a laser confocal microscope (Olympus). Cy5 fluorophore was attached to the end of miR29c during synthesis, as described in Section 2.1, and mixed with culture medium at 3% serum concentration. BMSCs were seeded in a 12‐well plate overnight, allowing adherence, before treatment with the aforementioned nanomaterial/medium mixture. After 12 h, the cells were fixed and stained with 4′,6‐diamidino‐2‐phenylindole (DAPI, Beyotime) and fluorescein isothiocyanate isomer I (FITC, Beyotime) solution. Finally, all samples were sealed with 10% (v/v) glycerol solution and examined under a laser confocal microscope to observe intracellular material uptake. Images were captured from selected fields of view.

### 2.6 Biocompatibility of stFNAs and stFNAs‐miR29c

Various concentrations of stFNAs and stFNAs‐miR29c solutions were prepared and mixed with culture medium at 3% serum concentration. Cells were evenly seeded and cultured overnight to allow adhesion. After washing with phosphate‐buffered saline (PBS), BMSCs were cultured for 12 h in medium containing either stFNAs or stFNAs‐miR29c. Subsequently, cell counting kit‐8 (CCK‐8) assay solution (MCE) was added, and after 1 h of incubation, the optical density (OD) values of BMSCs treated with different material concentrations were measured at 450 nm.

### 2.7 Protein level identification of relevant markers in BMSCs

The protein expression levels of relevant markers in BMSCs were determined using immunofluorescence and western blot assays. Following 12 h of treatment with various materials, rat BMSCs were induced for osteogenic differentiation using an Oricell induction kit. Cells were then fixed with paraformaldehyde at 4°C and treated with 0.5% Triton X‐100 solution for cell membrane permeabilisation. Samples were incubated with 5% goat serum for 1 h, followed by overnight incubation at 4°C with diluted primary antibodies. Subsequently, diluted secondary antibodies were applied for 1 h, with nuclei and cytoskeleton staining using DAPI and FITC. PBS was used to rinse the cells before and after applying these solutions. The slides were sealed with 10% (v/v) glycerol solution and observed under laser confocal microscopy. Total intracellular protein was extracted using a KeyGen Biotech kit. A 5 × SDS‐PAGE loading buffer (Beyotime) was added in a 4:1 ratio, and the samples were boiled and stored at −80°C. Proteins were separated by SDS‐PAGE, and then transferred to polyvinylidene difluoride (PVDF) membranes. Rapid blocking was performed for 15–20 min, followed by overnight incubation at 4°C with primary antibodies and incubation with secondary antibodies for 1 h at room temperature. Membranes were visualised using an ECL developer and captured by an enhanced chemiluminescence detection system (Bio‐Rad). Markers detected included osteogenesis‐related factors such as alkaline phosphatase (ALP), osterix (OSX), RUNX2 and osteopontin (OPN); Wnt pathway‐related factors (beta‐catenin, DKK1); and peroxisome proliferator‐activated receptor‐γ (PPAR‐GAMA), identified as an adipogenesis‐associated factor. All primary antibodies were obtained from HuaBio Co., Ltd., with the following catalogue numbers: anti‐ALP (ET1601‐21), anti‐OSX (ET1914‐47), anti‐RUNX2 (ET1612‐47), anti‐OPN (0806–6), anti‐PPAR‐GAMA (ET1702‐57), anti‐beta‐catenin (ET1601‐5) and anti‐DKK1 (ET1610‐63).

### 2.8 Gene level identification of relevant markers in BMSCs

Gene expression levels of osteogenesis and Wnt pathway markers in BMSCs were monitored using reserve transcription quantitative real‐time polymerase chain reaction (RT‐qPCR). Following 12 h of treatment with different nanomaterials, osteogenic differentiation of BMSCs was induced using an Oricell induction kit. RNA from BMSCs was extracted and purified using an RT‐qPCR kit (TaKaRa) and reverse‐transcribed into cDNA. mRNA expression levels of related markers were quantitatively analysed using a real‐time fluorescence quantitative PCR machine (Thermofisher). GAPDH was used as the internal reference for mRNA expression levels. All primers, listed in Supporting Information, Table S2, were synthesised by Sangon. Detected markers included osteogenesis‐related factors (*Alp*, *Runx2*, *Osx* and *Opn*), Wnt pathway‐related factors (*Beta‐catenin* and *Dkk1)* and the adipogenesis‐associated factor (*Ppar‐gama)*.

### 2.9 Identification of ALP activity in BMSCs

Following 12 h of treatment with various nanomaterials, rat BMSCs were induced for osteogenic differentiation using an Oricell induction kit. ALP activity in BMSCs was assessed after 7 days of osteogenic induction using an ALP detection kit, with ALP activity visible as a blue‐violet precipitate under the microscope.

### 2.10 Identification of calcium nodule deposition in BMSCs

After 12 h of treatment with different materials, osteogenesis in rat BMSCs was induced using the Oricell osteogenic differentiation induction kit. Osteogenic differentiation was further assessed by alizarin red staining, which precipitates calcium ions into an orange‐red complex.

### 2.11 Identification of lipid droplet expression levels in BMSCs

Following 12 h of treatment with various materials, BMSCs were induced for adipogenesis using the Oricell Adipogenic Differentiation Induction Kit and an oil red O staining experiment was used after 14 days to evaluate the adipogenesis ability of BMSCs.

### 2.12 Establishment of critical‐sized bone defects

The experiments commenced after 1‐week‐adaptive feeding of male SD rats, weighing 200–250 g. Following anaesthesia, a 2‐cm longitudinal incision was made to expose the surgical area fully. A circular bone drill created bilateral, symmetrical and full‐thickness critical‐sized bone defects (CSBDs) at the sagittal suture, with saline used for cooling throughout the procedure. Care was taken to preserve the dura mater's integrity to avoid brain tissue damage. The incision was sutured in layers using 5‐0 absorbable thread, and postoperative penicillin was administered intraperitoneally for three consecutive days to prevent infection. The experiment was approved by the Ethics Committee of West China Hospital of Stomatology, Sichuan University (WCHSIRB‐D‐2023‐319).

### 2.13 Sample collection

Drugs were locally injected into the cranial defects at a frequency of every other day. One‐ and 2‐month post‐administration, bone tissue, including the defect area, was excised using tissue scissors. Samples were trimmed with bone scissors and fixed in paraformaldehyde. To minimise the impact of prolonged fixation on subsequent fluorescence staining, samples were transferred to PBS after overnight fixation and stored at 4°C.

### 2.14 CT reconstruction and analysis

Micro‐computed tomography (micro‐CT) was utilised to evaluate the repair of CSBDs and the quality of new bone formation. This included a three‐dimensional reconstruction of the samples and assessment of bone volume fraction (BV/TV), trabecular number (Tb.N), trabecular thickness (Tb.Th) and trabecular separation (Tb.Sp). These quantitative indicators were statistically analysed.

### 2.15 Histological and immunofluorescence staining analysis

Samples were decalcified in ethylene diamine tetraacetic acid solution, with the solution changed every 3 days until a pin could easily pass through the bone tissue (approximately 4 weeks). Processes of paraffin embedding, sectioning, haematoxylin–eosin (H&E) and immunofluorescence staining were conducted. Observations were made using a microscope and slide scanner.

### 2.16 Statistical analysis

One‐way analysis of variance was employed for group comparisons. Each experimental group comprised a sample size of *n* ≥ 3.

## 3 RESULTS

### 3.1 Synthesis and characterisation of stFNAs and stFNAs‐miR29c

**FIGURE 1:**
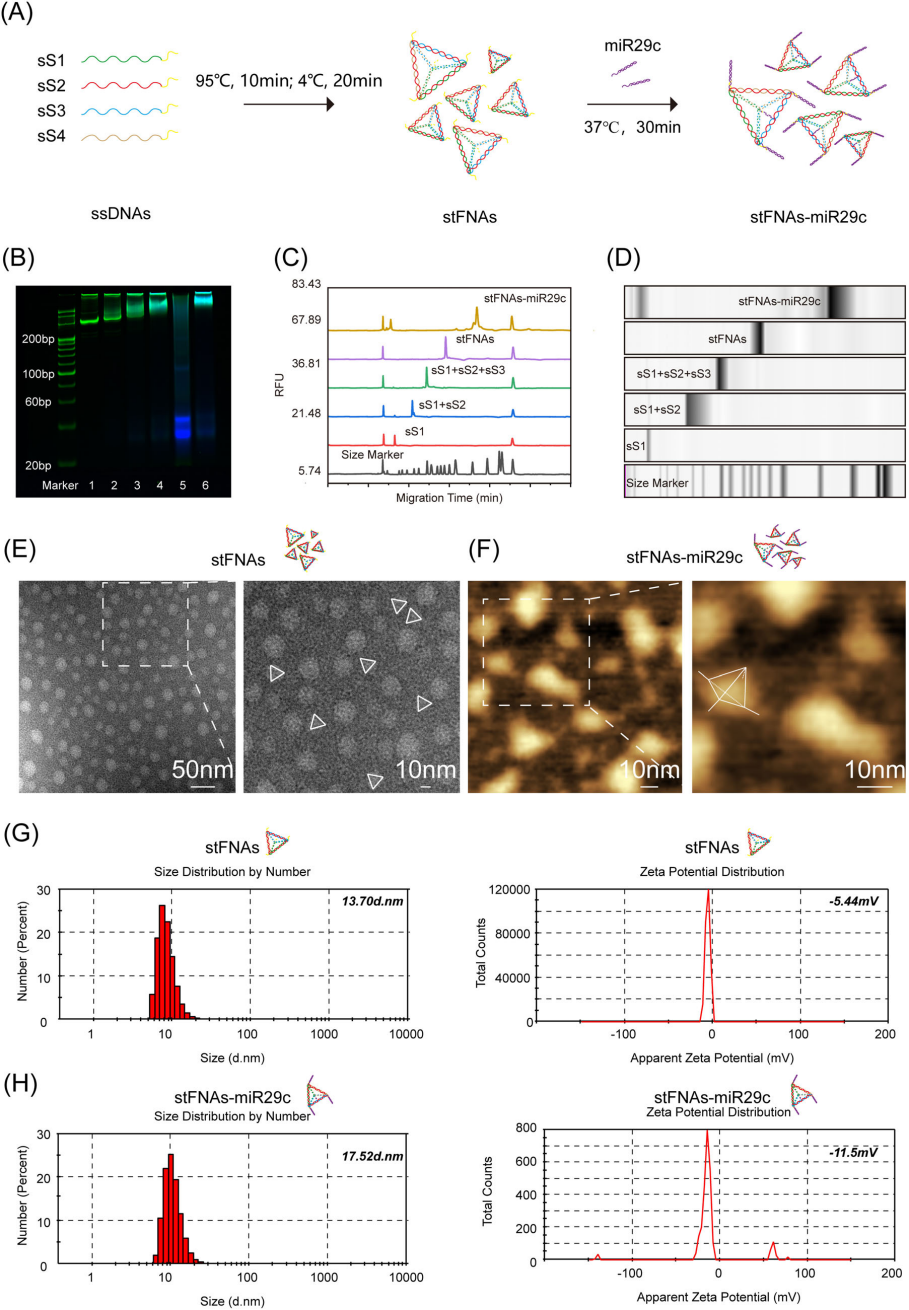
Synthesis and characterisation of stFNAs and stFNAs‐miR29c. (A) Schematic of the synthesis procedure. (B) PAGE results confirming successful synthesis (1: sS1; 2: sS1 + sS2; 3: sS1 + sS2 + sS3; 4: stFNAs; 5: miR29c and 6: stFNAs‐miR29c). (C) CE results indicating successful preparation. (D) Gel plots from CE. (E) TEM image displaying the tetrahedral structure of stFNAs. (F) AFM image showing miR29c structure. (G) Particle size and ζ potential of stFNAs. (H) Particle size and ζ potential of stFNAs‐miR29c. AFM, atomic force microscopy; TEM, transmission electron microscopy.

As illustrated in Figure 1A, we constructed stFNAs with sticky ends based on a previously established synthesis procedure, subsequently attaching miR29c to the four vertices of stFNAs through base complementary pairing, resulting in the stFNAs‐miR29c complex. The successful synthesis of this complex was confirmed by PAGE (Figure 1B). The gel lanes, from left to right, included the marker, sS1, sS1 + sS2, sS1 + sS2 + sS3, stFNAs, miR29c and stFNAs‐miR29c, with stFNAs‐miR29c exhibiting the slowest migration rate and highest molecular weight. The CE results, depicted in Figure 1C,D, were consistent with the PAGE findings, indicating that stFNAs‐miR29c had the longest migration time, suggesting a larger molecular weight compared to ssDNA and stFNAs alone. These results collectively affirmed the successful synthesis of stFNAs‐miR29c. TEM and AFM images, shown in Figure 1E,F, further validated the nanostructures of stFNAs and stFNAs‐miR29c, revealing the unique tetrahedral structure of stFNAs and the appended ‘small tail’ of miR29c. Additionally, DLS analysis, presented in Figure 1G,H, indicated that stFNAs had an approximately 13.7 d.nm particle size and a −5.44 mV ζ potential, whereas stFNAs‐miR29c exhibited a larger particle size (17.52 d.nm) and a lower ζ potential (−11.5 mV), attributable to the negatively charged miR29c.

### 3.2 Biological characteristics, mechanism and osteogenesis ability of stFNAs‐miR29c

**FIGURE 2:**
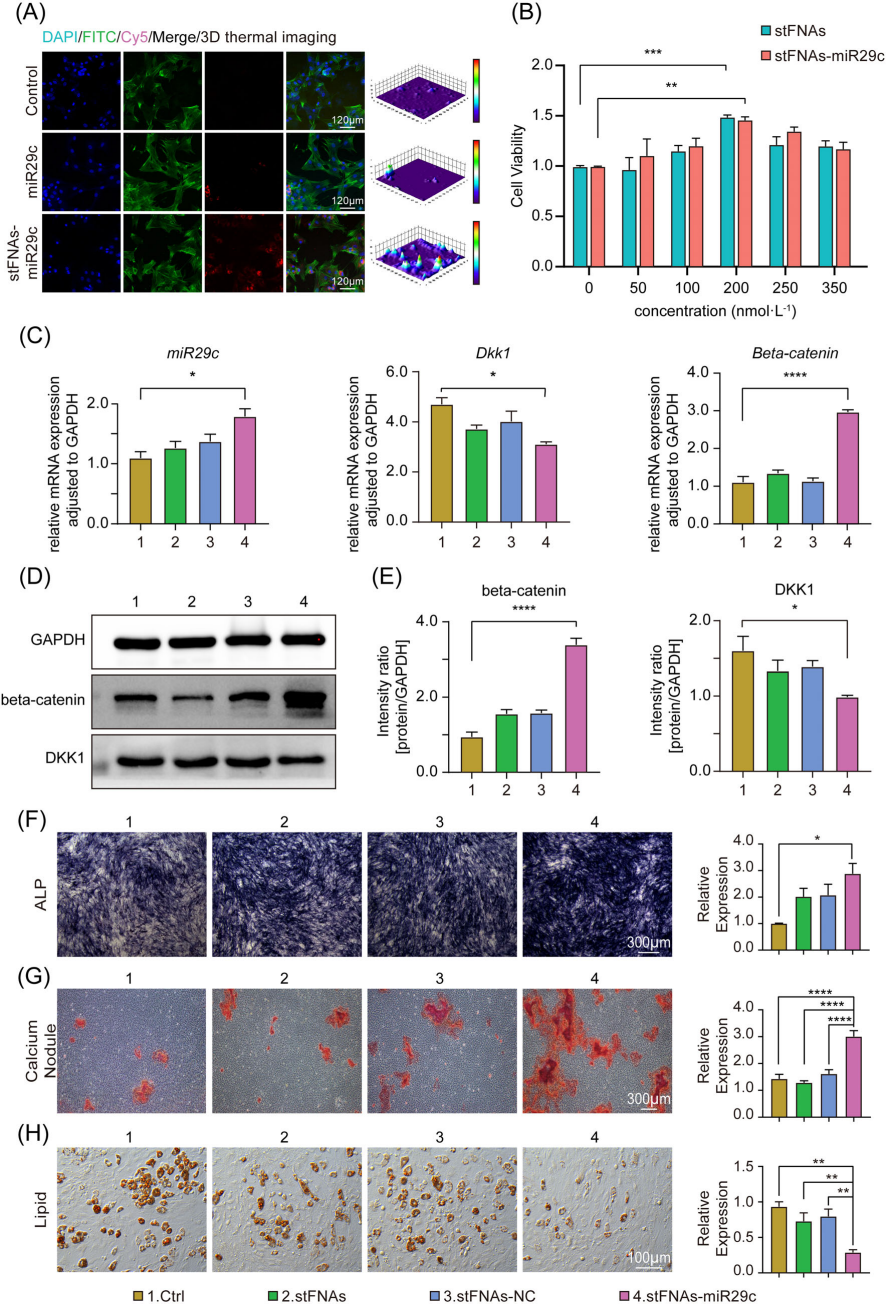
Biological characteristics, mechanism, and osteogenesis ability of stFNAs‐miR29c. (A) Uptake of stFNAs‐miR29c‐Cy5 and miR29c‐Cy5 by BMSCs after 12 h of treatment with stFNAs‐miR29c. (B) CCK‐8 assay results for various concentrations of stFNAs and stFNAs‐miR29c. (C) RT‐qPCR results for *miR29c*, *Dkk1* and *Beta‐catenin*. (D) Western blot results for beta‐catenin and DKK1. (E) Statistical analysis of Western blot results is shown in Figure 2D. (F) Results and statistical analysis of alkaline phosphatase staining 7 days after osteogenic induction. (G) Results and statistical analysis of alizarin red staining 21 days after osteogenic induction. (H) Results and statistical analysis of oil red O staining 14 days following adipogenesis induction. ANOVA, analysis of variance; CCK‐8, cell counting kit‐8; RT‐qPCR, reserve transcription quantitative real‐time polymerase chain reaction. ****p < 0.0001, ***p < 0.001, **p < 0.01, *p < 0.05 (ANOVA analysis, *n* ≥ 3).

Immunofluorescence and confocal microscopy were employed to investigate the endocytosis of stFNAs‐miR29c. Results depicted in Figure 2A demonstrated a significantly higher intracellular fluorescence intensity in BMSCs treated with stFNAs‐miR29c, indicating enhanced cellular uptake compared to miR29c alone. As evidenced in Figure 2B, both stFNAs and stFNAs‐miR29c exhibited the highest BMSCs proliferation activity at a concentration of 200 nM, according to CCK‐8 assays. These findings provided a robust basis for selecting the optimal concentration of stFNAs‐miR29c for BMSCs osteogenesis induction. Therefore, we chose 200 nM as the most appropriate concentration of stFNAs‐miR29c in vitro. We hypothesised that stFNAs‐miR29c might elevate the expression of osteogenesis‐related factors through the Wnt signalling pathway. Subsequent analyses of Wnt pathway‐related markers revealed, in Figure 2C,D, that stFNAs‐miR29c treatment significantly reduced mRNA and protein expression levels of DKK1, an inhibitory factor of the pathway, while markedly increasing the expression levels of beta‐catenin, a positive regulator. The Western blot statistical analysis in Figure 2E corroborated these findings, consistent with the RT‐qPCR results in Figure 2C. The differences in DKK1 and beta‐catenin expression between the stFNAs‐miR29c and Ctrl groups were statistically significant. In Figure 2F–H, staining assays evaluated the effects of stFNAs‐miR29c on osteogenesis and adipogenesis in BMSCs. Osteogenesis was induced in BMSCs 12 h post‐treatment with stFNAs‐miR29c, followed by detection of early osteogenic differentiation marker ALP using the BCIP/NBT alkaline phosphatase chromogenic kit after 7 days (Figure 2F), and late marker calcium nodule using alizarin red staining after 21 days (Figure 2G). These assays revealed significantly increased NBT formation and calcium nodule deposition in the stFNAs‐miR29c group. Quantitative analyses of ALP and alizarin red staining, as shown in Figure 2F,G, respectively, indicated a statistically significant difference compared to the Ctrl group. Following 14 days of adipogenesis induction, the stFNAs‐miR29c group displayed markedly reduced lipid droplet formation, significantly differing from the Ctrl group, as demonstrated in Figure 2H. These results further validated that stFNAs‐miR29c positively regulated BMSCs' osteogenesis and appeared to inhibit adipogenesis to a certain degree.

### 3.3 Expression of osteogenesis‐specific markers in the presence of stFNAs‐miR29c

**FIGURE 3:**
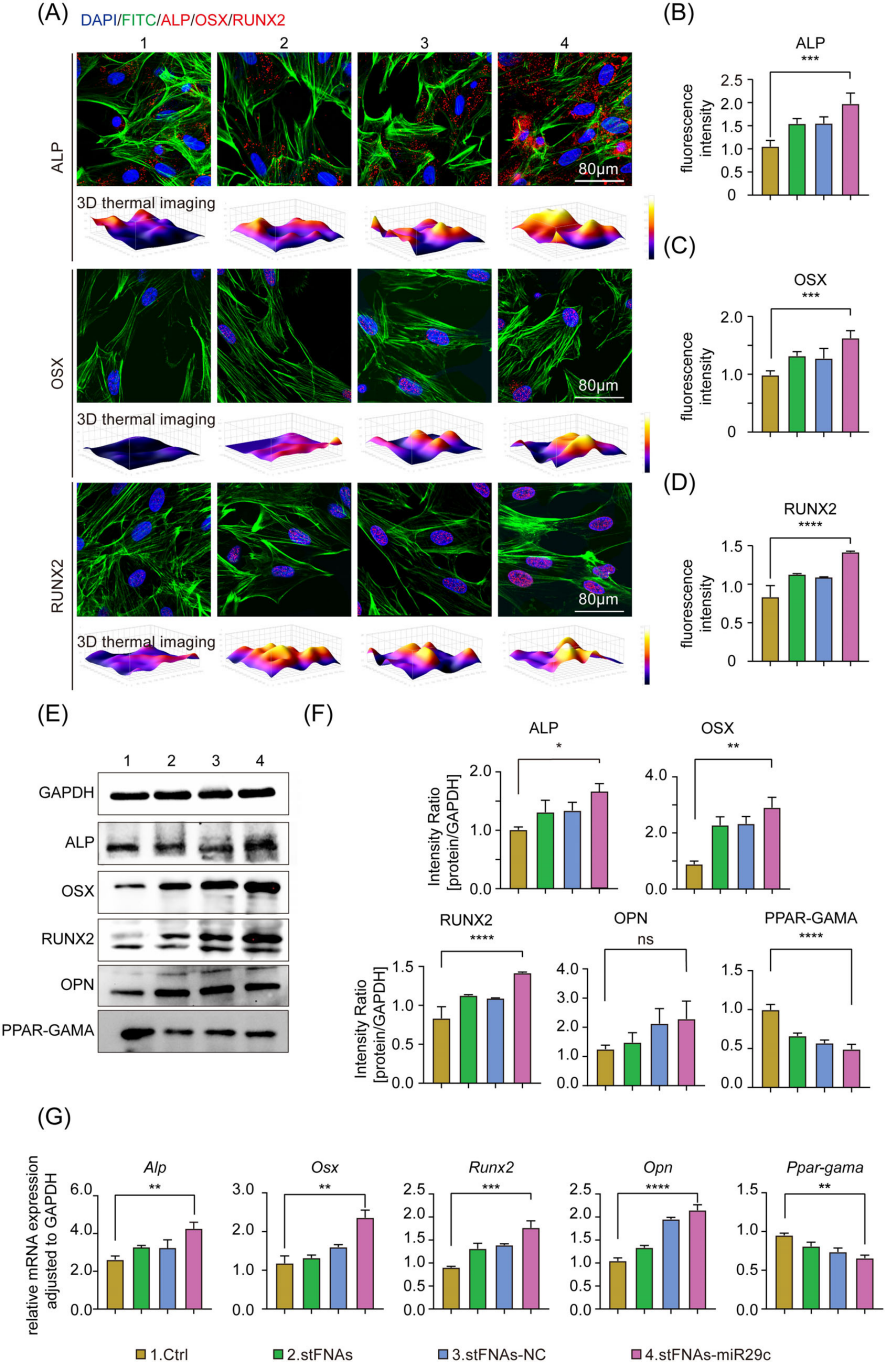
Expression of osteogenesis‐specific markers in the presence of stFNAs‐miR29c. (A) Immunofluorescence staining results for ALP, OSX, and RUNX2 after 7 days of osteogenic induction. (B) Statistical analyses of fluorescence staining results for ALP. (C) Statistical analyses of fluorescence staining results for OSX. (D) Statistical analyses of fluorescence staining results for RUNX2. (E) Western blot results for ALP, OSX, RUNX2, OPN post‐osteogenic induction and PPAR‐GAMA post‐adipogenic induction. (F) Statistical analysis of Western blot results. (G) RT‐qPCR findings for *Alp*, *Osx*, *Runx2*, and *Opn* post‐osteogenic induction and *Ppar‐gama* following adipogenic induction. ANOVA, analysis of variance; RT‐qPCR, reserve transcription quantitative real‐time polymerase chain reaction. ****p < 0.0001, ***p < 0.001, **p < 0.01, *p < 0.05, *ns*, no significance (ANOVA analysis, *n* ≥ 3).

Based on the findings in Figure 2, stFNAs‐miR29c was observed to enhance the osteogenesis process of BMSCs by upregulating the Wnt signalling pathway. Immunofluorescence staining, RT‐qPCR and western blot assays were utilised to assess the expression levels of specific markers (ALP, OSX, RUNX2, OPN and PPAR‐GAMA) associated with early, middle and late stages after osteogenesis induction when treated with stFNAs‐miR29c. Figure 3A demonstrates significantly increased expression of ALP, OSX and RUNX2 in the stFNAs‐miR29c group, indicated by the highest fluorescence intensity. Figure 3B–D present statistically significant differences between the stFNAs‐miR29c and Ctrl groups in the immunofluorescence staining experiments. These results were further supported by western blot and RT‐qPCR analyses (Figure 3E–G). Additionally, protein and mRNA expression levels of OPN, a late osteogenic differentiation marker, were found to be approximately double that of the Ctrl group (Figure 3F,G). In contrast, the protein and gene expression levels of adipogenesis‐specific marker PPAR‐GAMA in the stFNAs‐miR29c group were reduced to about half compared to the Ctrl group (Figure 3F,G). The overall results from Figures 2 and 3 suggested that stFNAs‐miR29c activated the Wnt signalling pathway by inhibiting DKK1 expression, thus promoting the expression of subsequent osteogenesis‐specific factors and enhancing BMSCs osteogenic differentiation. Additionally, stFNAs‐miR29c appeared to downregulate PPAR‐GAMA expression, thereby inhibiting adipogenesis in BMSCs, although the underlying mechanisms warranted further investigation.

### 3.4 Micro‐CT analysis of CSBDs repair with stFNAs‐miR29c

**FIGURE 4:**
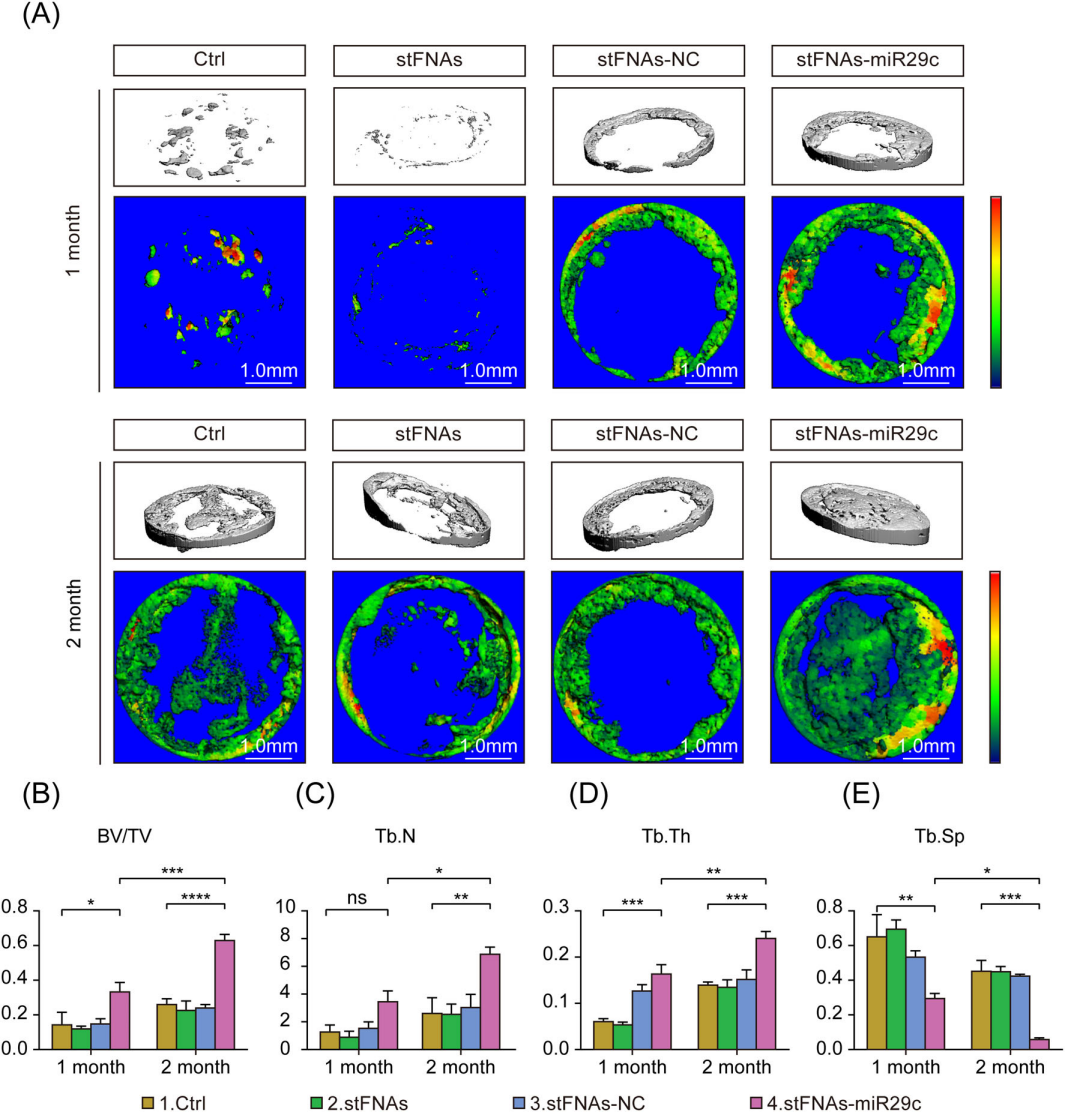
Micro CT analysis of CSBDs repair with stFNAs‐miR29c. (A) Three‐dimensional skull reconstruction and comparison of bone regeneration effects across groups. (B) Statistical analysis of BV/TV results. (C) Statistical analysis of Tb.N results. (D) Statistical analysis of Tb.Th results. (E) Statistical analysis of Tb.Sp results. ANOVA, analysis of variance; BV/TN, bone volume fraction; CSBD, critical‐sized bone defect; CT, computed tomography; Tb.N, trabecular number; Tb.Sp, trabecular separation; Tb.Th, trabecular thickness. ****p < 0.0001, ***p < 0.001, **p < 0.01, *p < 0.05, *ns*, no significance (ANOVA analysis, *n* ≥ 3).

Micro‐CT was utilised to observe and quantitatively analyse new bone tissue quality in CSBDs. Three‐dimensional skull reconstructions revealed initial sporadic new bone deposition around the defect, progressively forming a ring along the defect's boundary before filling towards the centre. As shown in Figure 4A, at 2 months, the stFNAs‐miR29c group exhibited extensive new bone formation, nearly encompassing the entire defect area, indicating substantial bone regeneration and repair effects. Figure 4B–E quantified the volume and density of new bone in the region of interest (ROI), showing significant increases in BV/TV, Tb.Th and Tb.N, and a marked decrease in Tb.Sp in the stFNAs‐miR29c group at 2 months. These findings suggested that stFNAs‐miR29c enhanced bone regeneration over catabolism in the defect area, resulting in superior bone quality compared to the control group, with more pronounced regeneration and reconstruction than at 1 month.

### 3.5 Histologic results of CSBDs repairment under the action of stFNAs‐miR29c

**FIGURE 5:**
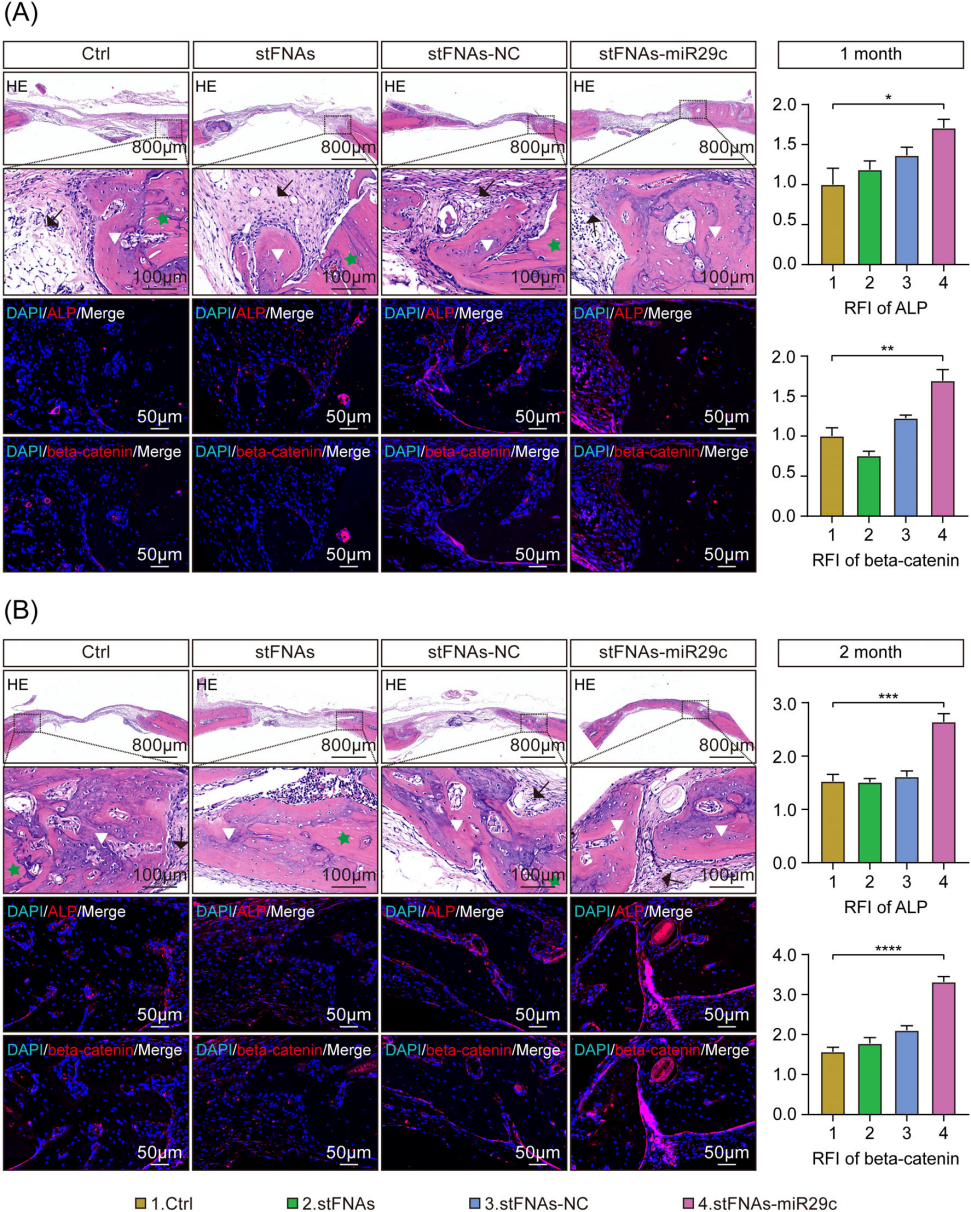
Histologic results of CSBDs repairment under the action of stFNAs‐miR29c. (A) H&E and immunofluorescence staining images with statistical analysis results of 1‐month administrated samples. (B) H&E and immunofluorescence staining images with statistical analysis results of 2‐month administrated samples. ANOVA, analysis of variance; CSBD, critical‐sized bone defect; H&E, haematoxylin and eosin. ****p < 0.0001, ***p < 0.001, **p < 0.01, *p < 0.05 (ANOVA analysis, *n* ≥ 3).

The bone regeneration and reconstruction capabilities of stFNAs‐miR29c were further assessed using H&E and immunofluorescence staining. As depicted in Figure 5A, at 1 month, all groups showed minimal new bone or osteoid formation, indicated by triangles, with collagen fibres and connective tissue predominating at this stage, as shown by arrows. Immunofluorescence staining of ALP and beta‐catenin proteins at 1 month revealed their highest expression in the stFNAs‐miR29c group, with significant differences compared to the control group (ALP, *p* < 0.05 and beta‐catenin, *p* < 0.01). In Figure 5B, noticeable bone regeneration and reconstruction were observed in all groups at 2 months, with the stFNAs‐miR29c group exhibiting the most significant new bone formation and markedly higher expression levels of ALP and beta‐catenin proteins than other groups, more pronounced than that at 1 month (ALP, *p* < 0.001 and beta‐catenin, *p* < 0.0001). This analysis provided a general understanding of local bone regeneration across groups through H&E staining at the tissue level and elucidated the mechanism and osteogenic microenvironmental modulation function of stFNAs‐miR29c in vivo through immunofluorescence staining at the microscopic cellular and protein levels.

## 4 DISCUSSION

The incidence of bone defects and fractures has been steadily increasing due to factors like trauma, inflammation and tumours.^31^ The prevalent clinical method of autologous bone grafting is hampered by limited donor bone volume, lengthy surgical procedures and considerable postoperative discomfort,^32^, ^33^ imposing significant burden and pain on patients.^34^ Many reported bone grafts lack effective osteo‐conductivity and osteo‐inductivity, resulting in subpar bone formation.^35^ However, the advancement and refinement of bone tissue engineering, an interdisciplinary field encompassing biology, engineering and materials science, have introduced biomolecular strategies to augment bone regeneration and repair. These strategies include stem cell therapies, extracellular matrix, growth factors and gene therapy,^36^ playing a pivotal role in tissue regeneration and repair.^34^

Specific miRNA molecules, notably miR29c, are crucial in the osteogenic differentiation of BMSCs, contributing to the repair of local bone tissue defects.^34^ MiR29c enhances the expression of osteogenesis‐specific proteins such as RUNX2 by activating the Wnt signalling pathway. Nevertheless, miR29c is plagued by poor stability and endocytosis.^37^ Existing delivery vectors, like viral transfection and liposomes, often face challenges of low delivery efficiency and biosafety concerns.^34^ Consequently, identifying a safe and efficient miRNA vector is imperative.

As a self‐assembled DNA tetrahedral structure, tFNAs demonstrate outstanding editability, biosafety and endocytosis ability^38^, ^39^ and are extensively employed in small molecule material delivery^38^, ^40^, ^41^, ^42^, ^43^ and tissue regeneration.^22^, ^44^, ^45^, ^46^, ^47^ Hence, we engineered tFNAs with sticky ends to bind miR29c via base complementary pairing, facilitating miR29c delivery and cell membrane penetration in vivo, thereby exerting intracellular biological functions, modulating the local osteogenic microenvironment, and enhancing bone regeneration and repair.

Material characterisation revealed that stFNAs‐miR29c could be successfully synthesised, exhibiting effective endocytosis, thereby transporting substantial quantities of miR29c into BMSCs and functioning accordingly. Furthermore, stFNAs‐miR29c demonstrated excellent biosafety and a certain ability to promote cell proliferation when administered at an appropriate concentration.

In vitro, at the cellular level, the osteogenic induction effect of stFNAs‐miR29c was analysed. Through immunofluorescence, RT‐qPCR, western blot and various staining experiments, it was evident that stFNAs‐miR29c notably upregulated the Wnt signalling pathway and increased the protein and gene expression levels of osteogenesis‐related factors (ALP, RUNX2, OSX and OPN). This, in turn, boosted the osteogenic differentiation capability of BMSCs,^48^, ^49^ enhancing alkaline phosphatase activity and mineralisation capacity of the BMSCs. Interestingly, under the influence of stFNAs‐miR29c, BMSC adipogenesis was suppressed, as evidenced by reduced PPAR‐GAMA expression and diminished lipid droplet formation.

In vivo, at the tissue level, we established a rat CSBD model. We observed that stFNAs‐miR29c significantly enhanced the regeneration of localised bone defects, particularly at 2 months, when cranial defects were nearly fully repaired. We conducted immunofluorescence staining experiments for ALP and beta‐catenin, linking tissue‐level outcomes to cellular and protein‐level mechanisms. These experiments showed that beta‐catenin, associated with the Wnt signalling pathway, and ALP, an osteogenesis‐specific protein, were markedly increased in the stFNAs‐miR29c group. This supported the conclusion that stFNAs‐miR29c effectively elevated the expression of key osteogenic factors through the Wnt signalling pathway, thus guiding BMSCs towards osteoblastic differentiation and enhancing the deposition of local mineralised nodules. This process leads to functional new bone formation and bone tissue reconstruction at the tissue level.

In summary, stFNAs‐miR29c served as an efficient nano nucleic acid carrier, enhancing miR29c stability, reducing degradation during delivery and improving endocytosis, thereby increasing intracellular miR29c concentration and enhancing its biological functions. Notably, stFNAs did not interfere with the function of miR29c. Mechanically, stFNAs‐miR29c modulated the local osteogenic microenvironment and promoted bone regeneration by activating the classical Wnt pathway and enhancing the expression of proteins like RUNX2 and ALP.

However, this study presented some limitations, notably that stFNAs‐miR29c required continuous administration every other day to achieve optimal osteogenic effects. Future research could focus on improving this nanocomposite's structure, reducing its degradation rate and expanding its application in long‐term tissue defects, including CSBDs, as a direction for nano nucleic acid carriers.

## 5 CONCLUSION

In this study, we developed a novel miRNA nano‐nucleic acid vector, stFNAs‐miR29c, for treating CSBDs. It effectively overcame the issues of miR29c's poor stability and endocytosis ability, also enhancing its transport efficiency and biological function. Mechanically, stFNAs‐miR29c successfully activated the Wnt pathway and upregulated the expression of RUNX2 and other osteogenesis‐specific factors, thus promoting alkaline phosphatase activity, increasing mineralised nodule deposition and achieving optimal bone regeneration in CSBDs. Further exploration and optimisation of this system are essential for its broader application and promising prospects in tissue engineering.

## AUTHOR CONTRIBUTIONS

**Jiafei Sun** and **Xingyu Chen:** performed the experiments. **Yunfeng Lin** and **Xiaoxiao Cai:** supervised the experiments.

## CONFLICT OF INTEREST STATEMENT

The authors declare no conflicts of interest.

## Supporting information


**Table S1.** Sequences of every ssDNA and miR29c with sticky end.
**Table S2.** Primers for RT‐qPCR.

## Data Availability

All data are available upon reasonable request from the corresponding author with the publication.
