# Supplementary material for: MicroRNA‐29c‐tetrahedral framework nucleic acids: Towards osteogenic differentiation of mesenchymal stem cells and bone regeneration in critical‐sized calvarial defects
Source: Cell Prolif. 2024 Feb 27;57(7):e13624. doi: 10.1111/cpr.13624 (PMC11216942; doi:10.1111/cpr.13624)
Supplement: Supplementary file 1 — Table S1. Sequences of every ssDNA and miR29c with sticky end. Table S2. Primers for RT‐qPCR. [file CPR-57-e13624-s001.docx]

Supporting Information

**Microrna-29c-Tetrahedral Framework Nucleic Acids: Towards Osteogenic Differentiation of Mesenchymal Stem Cells and Bone Regeneration in Critical Sized Calvarial Defects**

Jiafei Sun^1,2^, Xingyu Chen^1,2^, Yunfeng Lin^1,2^, and Xiaoxiao Cai^1,2, *^

^1^State Key Laboratory of Oral Diseases, National Center for Stomatology, National Clinical Research Center for Oral Diseases, West China Hospital of Stomatology, Sichuan University, Chengdu 610041, China

^2^Sichuan Provincial Engineering Research Center of Oral Biomaterials, Chengdu, Sichuan 610041, China

^*^Corresponding Author

Xiaoxiao Cai

E-mail: xcai@scu.edu.cn

^1^ State Key Laboratory of Oral Diseases, National Center for Stomatology, National Clinical Research Center for Oral Diseases, West China Hospital of Stomatology, Sichuan University, Chengdu 610041, China

**Tables**

| ssDNA | Sequences (from 5’ to 3’) |
| --- | --- |
| sS1 | TTGACCTGTGAATTATTTATCACCCGCCATAGTAGACGTATCACCAGGCAGTTGAGACGAACATTCCTAAGTCTGAA |
| sS2 | TTGACCTGTGAATTACATGCGAGGGTCCAATACCGACGATTACAGCTTGCTACACGATTCAGACTTAGGAATGTTCG |
| sS3 | TTGACCTGTGAATTACTACTATGGCGGGTGATAAAACGTGTAGCAAGCTGTAATCGACGGGAAGAGCATGCCCATCC |
| sS4 | TTGACCTGTGAATTACGGTATTGGACCCTCGCATGACTCAACTGCCTGGTGATACGAGGATGGGCATGCTCTTCCCG |
| miR29c | Sense: uucacaggucaauagcaccauuugaaaucgguua |
|  | Antisense：uaaccgauuucaaauggugcua |

**Table S1.** Sequences of every ssDNA and miR29c with sticky end.

| RNA | Sequences |
| --- | --- |
| *miR29c* | Forward primer: 5’-ACACAGGCTGACCGATTTCT-3’  Forward primer: 5’-TGGTGCTAGACAAAAACAGACT-3’ |
| *Dkk1* | Forward primer: 5’-TCCCATCCCTGCTTTGT-3’  Reverse primer: 5’-GCGGAACCTCAACTTCG-3’ |
| *Beta-catenin* | Forward primer: 5’-GGAAAGCAAGCTCATCATTCTG-3’  Reverse primer: 5’-AGTGCCTGCATCCCACCAGCTT-3’ |
| *Runx2* | Forward primer: 5’-GAACCAAGAAGGCACAGAC-3’  Reverse primer: 5’-AATGCGCCCTAAATCACTG-3’ |
| *Ppar-gama* | Forward primer: 5’-GTCTCACAATGCCATCAGGTTT-3’  Reverse primer: 5’-TTCAGCTGGTCGATATCACTGG-3’ |
| *Alp* | Forward primer: 5’-GGACAATGAGATGCCGCCAGAG-3’  Reverse primer: 5’-CCGAGAGGGAAGGGTCAGTCAG-3’ |
| *Opn* | Forward primer: 5’-CCAGCCAAGGACCAACTAC-3’  Reverse primer: 5’-AGTGTTTGCTGTAATGCGCC-3’ |
| *Osx* | Forward primer: 5’-GGAGGCACAAAGAAGCCATA-3’  Reverse primer: 5’-GGGAAAGGGTGGGTAGTCAT-3’ |
| *Gapdh* | Forward primer: 5’-ACAGCAACAGGGTGGTGGAC-3’  Reverse primer: 5’-TTTGAGGGTGCAGCGAACTT-3’ |

**Table S2.** Primers for RT-qPCR.
